# Supplementary material for: Mechanism-Driven and Clinically Focused Development of Botanical Foods as Multitarget Anticancer Medicine: Collective Perspectives and Insights from Preclinical Studies, IND Applications and Early-Phase Clinical Trials
Source: Cancers (Basel). 2023 Jan 23;15(3):701. doi: 10.3390/cancers15030701 (PMC9913787; doi:10.3390/cancers15030701)
Supplement: Supplementary file 1 [file cancers-15-00701-s001.zip › cancers-2063036-supplementary.pdf]

Supplement Table S1. List of anticancer foods with the bioactive molecules, mechanism of action and function endpoints.

Food	Functional Molecules	Targets and Mechanism of Action	Level of Evidence	Functional Endpoints	References
**Edible Mushroom**	B-glucans	TLRs0Myd88-NFκB-TNFα/IL6/IL12 Dectin-Syk-NLRP3-IL1β*ǂơ	Clinical, *in vitro,* and *in vivo* studies	Prime innate & adaptive immune cells	[S1 – S5, S57]
		MDSCs, M2 Macrophage*ǂơ	Clinical, *in vitro,* and *in vivo* studies	Reduce immunosuppressive cells	[S6]
	Conjugated Linoleic acid	Inhibitor aromatase, 5α-reductase and androgen receptor*ǂ	*In vitro* and *in vivo* studies	Inhibit E2/DHT formation Inhibit AR/PSA production	[S4, S5]
	Phytosterols (Tocopherols)	Inhibit tyrosinase*	*In vitro* studies	Suppress cell growth	[S4, S5]
	Ergosterol	Signaling molecules from Fox03: ╧ Fas, FasL, BimL & SimS*	*In vitro* studies	Inhibit cell growth	[S2, S4, S7]
	Flavonoids (myricetin, catechin)	Potent immune stimulants Protect against free radical damage*	*In vitro* studies	Promote immune response, disrupte redox homeostasis	[S2, S4, S56]
	Food Extract	Suppress prostaglandin E2, Ki67, DNA topoisomerase 2α*ǂ	*In vitro* and *in vivo* studies	Inhibit cell growth	[S8, S9]
**Broccoli**	Flavonoid (Kaempferol)	╧ Nfr2 transcriptional pathway*	*In vitro* studies	Eliminate redox reaction	[S29, S30]
		Inhibit NF-κB pathway*	*In vitro* studies	Decrease inflammation and redox activities	[S29, S30]
		Activates mitogen-activated protein kinase (MAPK) signaling pathway*	*In vitro* studies	Inhibit cell growth	[S29, S30]
		↓ VEGF expression/ ╤HIF-1α* G2/M phase cell cycle arrest* ╤ epithelial-mesenchymal transition (EMT)-related markers* ╤ PI3K/protein kinase B signaling pathways* ╤ ER signaling pathway*	*In vitro* studies	Obstruct redox activities	[S29, S30]
		AITC-SiQDs, nanoscale system, disrupt ROS homeostasis*	*In vitro* studies	Obstruct redox activities	[S29, S30]
	Allyl isothiocyanate	Activate Nrf2 ǂơ	Clinical trial and *in vivo* studies	Eliminate redox reaction	[S31]
	Sulforaphane	Target DNA methyltransferases (DNMTs), histone deacetyltransferases (HDACs) & noncoding RNAs*	*In vitro* studies	Epigenetics, cell cycle arrest	[S32]
	Glutathione	╧ antioxidant genes, such as Gclc, Gclm, heme oxygenase-1 (Hmox1), and NADPH quinone oxidoreductase-1 (Nqo1)*	*In vitro* studies	Disrupt redox homeostasis resulting in higher immune activities	[S33, S34]
	Allyl isothiocyanate nanoparticles	Inhibit TNF-α, IL-6, NO & iNOS production*	*In vitro* studies	cell growth inhibition, anti-inflammation, and redox activity suppression	[S35]
**Garlic**	Amino acids (Alliin, Allicin)	G2/M arrest* ╧ Bax & FasL expressions ǂ ╧ caspase-3, -8, & -9*	*In vivo* and *in vitro* studies	Inhibit cell growth	[S25. S26]
	Diallyl trisulfide (DATS)	↓ G1, M, & S phases *ǂ ↑ Caspase-3 activity *ǂ Sustain cyclin B1 expression *ǂ Inhibit spindle formation *ǂ	*In vivo* and *in vitro* studies	Inhibit cell growth	[S27]
	Saponins	Inhibit intracellular ROS* ↓ γH2AX, prevent H2O2-induced DNA damage* ↑ antioxidant enzyme heme oxygenase-1 (HO-1)* Activate Nrf2/HO-1 pathway*	*In vitro* studies	Eliminate redox activities	[S28]
	Flavonoid (Kaempferol)	╧ Nfr2 transcriptional pathway*	*In vitro* studies	Eliminate redox reaction	[S29, S30]
		Inhibit NF-κB pathway*	*In vitro* studies	Decrease inflammation and redox activities	[S29, S30]
		Activates mitogen-activated protein kinase (MAPK) signaling pathway* ↓ VEGF expression/ ╤HIF-1α*	*In vitro* studies	Inhibit cell growth	[S29, S30]

		G2/M phase cell cycle arrest*<br>╤ epithelial-mesenchymal transition (EMT)-related markers*<br>╤ PI3K/protein kinase B signaling pathways*			
**Berries**	Quercetin	╤ cyclin B1 & CDK1*<br>Inhibit topo II activity ǂ	*In vivo* and *in vitro* studies	Inhibit cell growth	[S49]
		DNA damage via ROS generation*	*In vitro* studies	Low amounts inhibit cell cycle progression but not induce cell death	[S49]
	Anthocyanin	Activate caspase 3-pathways*<br>↓ cell proliferation*<br>↓ PI3K/AKT & NFκB activation & phosphorylation *ǂ<br>↓ Ki-67 expression, ↑ caspase-3 expression/apoptosis ǂ<br>↓ AKT & p65 NF-κB signaling proteins ǂ	*In vivo* and *in vitro* studies	Inhibit cell growth	[S50 – S53]
		↑ anti-inflammatory cytokines in serum (MIG, IP-10, IL-12, IL-2, TNF-α)*	*In vitro* studies	Anti-inflammatory	[S54]
	Food extract (Jamun, blueberry)	Inhibit cell growth/proliferation, ↑ apoptosis *ǂ	*In vivo* and *in vitro* studies	Inhibit cell growth	[S53, S55]
**Pomegranate**	Anthocyanidins (Delphinidin, Cyanidin, Pelargonidin)	DNA damage in cancer cells via ROS generation*	*In vitro* studies	Oxidative stress and inflammation	[S10]
		↓ VEGF*	*In vitro* studies	Inhibit cell growth	[S11]
	Polyphenol (Ellagitannin, Ellagic Acid)	↓ specificity protein (Sp) transcription factors & Sp-related genes *ǂ	*In vivo* and *in vitro* studies	Inhibit cell growth	[S12]
		↓ miR-27a*ǂ, ╤ miRNA0155*	*In vivo* and *in vitro* studies	Regulate gene expression	[S12]
		╤ cyclin A, B1, ╧ cyclin E*	*In vitro* studies	Inhibit cell cycle	[S11]
		Inhibit AKT activity, NF-κB activation, and COX-2 expression*	*In vitro* studies	Oxidative stress and inflammation	[S13]
	Metabolite (Urolithin A & B)	Reduces IL-6, IL-1β, NOS2*	*In vitro* studies	Disrupt redox homeostasis	[S11, S14, S15]
		Inhibit aromatase activity*	*In vitro* studies	Inhibit E2 formation	[S11, S14, S15]
**Tomato**	Flavonoid (kaempferol, quercetin, naringenin)	Induce autophagy*	*In vitro* studies	Inhibit cell growth	[S17, S36]
	Carotenoid (Lycopene, Phytoene, Phytofluene, α- and β-carotene, β0cryptoxanthin, Lutein, Zeaxanthin)	Activate antioxidant enzymes (glutathione, glutathione peroxidase, & glutathione-S-transferase) ǂ (Epre/ARE) activation*	*In vivo* and *in vitro* studies	Disrupt redox homeostasis	[S36 – S39]
		Inhibit ERK phosphorylation*	*In vitro* studies	Inhibit cell growth	[S36 – S39]
		Androgen receptor inhibition*	*In vitro* studies	Inhibit AR activity	[S36 – S39]
**Walnut**	Polyphenol (Ellagitannin)	↓ cyclin B1 protein*	*In vitro* studies	Inhibit cell growth	[S40]
	Vitamins (Tocopherol, Niacin)	╧ PPAR activity*	*In vitro* studies	Inhibit cell growth	[S41]
	Flavonoid (quercetin, catechin)	Induce autophagy*	*In vitro* studies	Inhibit cell growth	[S17]
	Phytosterol (stigmasterol, campsterol, sitosterol)	Activate ER-mitochondrial axis to induce cell death*<br>╤ TNF-α & VEGFR-2*	*In vitro* studies	Inhibit cell growth	[S42, S43]
	Juglone	Enhance the level of Fox03a & Fox01 in cells*<br>Induce G2/M phase cell cycle arrest*	*In vitro* studies	Disrupt the DNA replication and cell cycle division	[S44]
		Activate JNK pathway & ROS-mediated p38 to initiate autophagy*	*In vitro* studies	Inhibit cell growth	[S44]
	Metabolites (Urolithins-A, -C, & IsoUro-A)	╤ mRNA & protein level in PSA & AR*	*In vitro* studies	Reduce PSA & AR activities	[S45]

		↓ Bcl-2 protein levels*	*In vitro* studies	Inhibit cell growth, induce apoptosis	[S45]
**Ginger**	Paradol	↓ EGFR protein expression*<br>Inactivate PI3K/AKT signaling*	*In vitro* studies	Inhibit cell growth	[S46, S47]
	Zingiberene	Damage microtubules*	*In vitro* studies	Inhibit cell growth	[S46]
	1-monolinolein	╤ NF-κB activation*<br>↑ caspase-3/7 activation for apoptosis*	*In vitro* studies	Inhibit cell growth	[S45, S48]

Note: ǂ in animal model; * in cell line; ơ clinical trial; ↑ increase; ↓ decrease; ╤ down-regulate; ╧ up-regulate.

Comment: The biological activities being studied are mainly from *in vitro* models and that no references to animal models have shown to reduce the growth of xenografted tumors or PDX grafts, but the targets and mechanism of action are sometimes both observed and corresponded to each other in both *in vitro* and *in vivo* studies. Although clinical trials do not specify that the botanical foods are proven to have anti-cancer activities, some trials have shown that patients receiving botanical foods and/or botanical food agents along with chemotherapy have prolonged the cancer patient's survival (e.g., references S1 & S57). Many clinical trials could not conclude that botanical foods do reduce tumor growth, as they claim a larger sample size is needed for a higher level of evidence.

References

S1. Friedman M. Mushroom Polysaccharides: Chemistry and Antiobesity, Antidiabetes, Anticancer, and Antibiotic Properties in Cells, Rodents, and Humans. *Foods*. **2016**;5(4):80. doi:10.3390/foods5040080

S2. You S. Mushrooms as Functional and Nutritious Food Ingredients for Multiple Applications. *ACS Food Science & Technology*. **2022** 2 (8), 1184-1195

S3. "Shiitake Mushroom." *Memorial Sloan Kettering Cancer Center*, 8 June 2020, Available at: www.mskcc.org/cancer-care/integrative-medicine/herbs/shiitake-mushroom.

S4. Valverde ME, Hernández-Pérez T, Paredes-López O. Edible mushrooms: improving human health and promoting quality life. *Int J Microbiol*. **2015** :376387. doi:10.1155/2015/376387

S5. Vamanu E. Antioxidant properties of mushroom mycelia obtained by batch cultivation and tocopherol content affected by extraction procedures. *Biomed Res Int*. **2014**. 974804. doi:10.1155/2014/974804

S6. Albeituni SH, Ding C, Liu M, et al. Yeast-Derived Particulate β-Glucan Treatment Subverts the Suppression of Myeloid-Derived Suppressor Cells (MDSC) by Inducing Polymorphonuclear MDSC Apoptosis and Monocytic MDSC Differentiation to APC in Cancer. *J Immunol*. **2016**;196(5):2167-2180. doi:10.4049/jimmunol.1501853

S7. Li X, Wu Q, Xie Y, et al. Ergosterol purified from medicinal mushroom Amauroderma rude inhibits cancer growth in vitro and in vivo by up-regulating multiple tumor suppressors. *Oncotarget*. **2015**;6(19):17832-17846. doi:10.18632/oncotarget.4026

S8. Lynn S. Adams, Shiuan Chen, Sheryl Phung, Xiwei Wu & Lui Ki. White Button Mushroom (Agaricus Bisporus) Exhibits Antiproliferative and Proapoptotic Properties and Inhibits Prostate Tumor Growth in Athymic Mice, *Nutrition and Cancer*, **2008**. 60:6, 744-756, DOI: 10.1080/01635580802192866

S9. Wang X, Ha D, Mori H, Chen S. White button mushroom (Agaricus bisporus) disrupts androgen receptor signaling in human prostate cancer cells and patient-derived xenograft. *J Nutr Biochem*. **2021**;89:108580. doi:10.1016/j.jnutbio.2020.108580.

S10. Farhan M, Rizvi A, Ali F, Ahmad A, Aatif M, Malik A, Alam MW, Muteeb G, Ahmad S, Noor A and Siddiqui FA. Pomegranate juice anthocyanidins induce cell death in human cancer cells by mobilizing intracellular copper ions and producing reactive oxygen species. *Front. Oncol.* **2022**.12:998346. doi: 10.3389/fonc.2022.998346.

S11. Larrosa M, Tomás-Barberán FA, Espín JC. The dietary hydrolysable tannin punicalagin releases ellagic acid that induces apoptosis in human colon adenocarcinoma Caco-2 cells by using the mitochondrial pathway. *J Nutr Biochem*. **2006** Sep;17(9):611-25. doi: 10.1016/j.jnutbio.2005.09.004.

S12. Banerjee N, Talcott S, Safe S, Mertens-Talcott SU. Cytotoxicity of pomegranate polyphenolics in breast cancer cells in vitro and vivo: potential role of miRNA-27a and miRNA-155 in cell survival and inflammation. *Breast Cancer Res Treat*. **2012.** 136(1):21-34. doi: 10.1007/s10549-012-2224-0.

S13. Mukherjee, Sudeshna et al. "Pomegranate Polyphenols Attenuate Inflammation and Hepatic Damage in Tumor-Bearing Mice: Crucial Role of NF-κB and the Nrf2/GSH Axis." *The Journal of nutritional biochemistry* vol. **2021**. 97: 108812. doi:10.1016/j.jnutbio.2021.108812

S14. Adams LS, Zhang Y, Seeram NP, Heber D, Chen S. Pomegranate ellagitannin-derived compounds exhibit antiproliferative and antiaromatase activity in breast cancer cells in vitro. *Cancer Prev Res (Phila)*. **2010**;3(1):108-113. doi: 10.1158/1940-6207.CAPR-08-0225

S15. Nallanthighal S, Elmaliki KM, Reliene R. Pomegranate extract alters breast cancer stem cell properties in association with inhibition of epithelial-to-mesenchymal transition. *Nutr Cancer*. 2017; 69:1088-98. doi: 10.1080/01635581.2017.1359318

S16. Chikara S, Nagaprashantha LD, Singhal J, Horne D, Awasthi S, Singhal SS. Oxidative stress and dietary phytochemicals: Role in cancer chemoprevention and treatment. *Cancer Lett*. **2018**. 28; 413:122-134. doi: 10.1016/j.canlet.2017.11.002.

S17. Wang P, Gao C, Wang W, et al. Juglone induces apoptosis and autophagy via modulation of mitogen-activated protein kinase pathways in human hepatocellular carcinoma cells. *Food Chem Toxicol*. **2018**;116(Pt B):40-50. doi:10.1016/j.fct.2018.04.004

S18. Yun Wang, Kai Woo Lee, Franky L. Chan, Shiuan Chen, Lai K. Leung, The Red Wine Polyphenol Resveratrol Displays Bilevel Inhibition on Aromatase in Breast Cancer Cells, Toxicological Sciences, Volume 92, Issue 1, July 2006, Pages 71–77, https://doi.org/10.1093/toxsci/kfj190

S19. Kaur M, Agarwal C, Agarwal R. Anticancer and cancer chemopreventive potential of grape seed extract and other grape-based products. *J Nutr*. **2009** Sep;139(9):1806S-12S. doi: 10.3945/jn.109.106864.

S20. Lu J, Zhang K, Chen S, Wen W. Grape seed extract inhibits VEGF expression via reducing HIF-1alpha protein expression. *Carcinogenesis*. **2009**;30(4):636-644. doi:10.1093/carcin/bgp009

S21. Wen W, Lu J, Zhang K, Chen S. Grape seed extract inhibits angiogenesis via suppression of the vascular endothelial growth factor receptor signaling pathway. *Cancer Prev Res (Phila)*. **2008**;1(7):554-561. doi:10.1158/1940-6207.CAPR-08-0040

S22. Dutta A, Chakraborty A. Cinnamon in Anticancer Armamentarium: A Molecular Approach. *J Toxicol*. **2018**. 29;2018:8978731. doi: 10.1155/2018/8978731.

S23. Sadeghi S, Davoodvandi A, Pourhanifeh MH, Sharifi N, ArefNezhad R, Sahebnasagh R, Moghadam SA, Sahebkar A, Mirzaei H. Anti-cancer effects of cinnamon: Insights into its apoptosis effects. *Eur J Med Chem*. **2019**. 15;178:131-140. doi: 10.1016/j.ejmech.2019.05.067.

S24. Gopalakrishnan S, Ismail A. Aromatic monophenols from cinnamon bark act as proteasome inhibitors by upregulating ER stress, suppressing FoxM1 expression, and inducing apoptosis in prostate cancer cells. *Phytother Res*. **2021**. 35(10):5781-5794. doi: 10.1002/ptr.7236.

S25. Sarvizadeh M, Hasanpour O, Naderi Ghale-Noie Z, et al. Allicin and Digestive System Cancers: From Chemical Structure to Its Therapeutic Opportunities. *Front Oncol*. **2021**; 11:650256. doi:10.3389/fonc.2021.650256

S26. Sarvizadeh M, Hasanpour O, Naderi Ghale-Noie Z, Mollazadeh S, Rezaei M, Pourghadamyari H, Masoud Khooy M, Aschner M, Khan H, Rezaei N, Shojaie L and Mirzaei H (2021) Allicin and Digestive System Cancers: From Chemical Structure to Its Therapeutic Opportunities. *Front. Oncol.* **2011**.11:650256. doi: 10.3389/fonc.2021.650256

S27. Hosono T, Fukao T, Ogihara J, et al. Diallyl trisulfide suppresses the proliferation and induces apoptosis of human colon cancer cells through oxidative modification of beta-tubulin. *J Biol Chem*. **2005**;280(50):41487-41493. doi:10.1074/jbc.M507127200

S28. Kang JS, Kim SO, Kim GY, et al. An exploration of the antioxidant effects of garlic saponins in mouse-derived C2C12 myoblasts. *Int J Mol Med*. **2016**;37(1):149-156. doi:10.3892/ijmm.2015.2398

S29. Imran M, Salehi B, Sharifi-Rad J, Aslam Gondal T, Saeed F, Imran A, Shahbaz M, Tsouh Fokou PV, Umair Arshad M, Khan H, Guerreiro SG, Martins N, Estevinho LM. Kaempferol: A Key Emphasis to Its Anticancer Potential. *Molecules*. **2019.**19;24(12):2277. doi: 10.3390/molecules24122277.

S30. Lee GA, Choi KC, Hwang KA. Kaempferol, a phytoestrogen, suppressed triclosan-induced epithelial-mesenchymal transition and metastatic-related behaviors of MCF-7 breast cancer cells. *Environ Toxicol Pharmacol*. **2017**;49:48-57. doi:10.1016/j.etap.2016.11.016

S31. Liu, P., Behray, M., Wang, Q. *et al.* Anti-cancer activities of allyl isothiocyanate and its conjugated silicon quantum dots. *Sci Rep*. **2018**. 8, 1084. doi.org/10.1038/s41598-018-19353-7

S32. Su X, Jiang X, Meng L, Dong X, Shen Y, Xin Y. Anticancer Activity of Sulforaphane: The Epigenetic Mechanisms and the Nrf2 Signaling Pathway. *Oxid Med Cell Longev*. **2018**; 5438179. doi:10.1155/2018/5438179

S33. Peng X, Urso M, Balvan J, Masarik M, Pumera M. Self-propelled magnetic dendrite-shaped microrobots for photodynamic prostate cancer therapy. *Angew Chem Int Ed Engl*. **2022**. 30 doi: 10.1002/anie.202213505.

S34. Melnikov F, Botta D, White CC, et al. Kinetics of Glutathione Depletion and Antioxidant Gene Expression as Indicators of Chemical Modes of Action Assessed in Vitro in Mouse Hepatocytes with Enhanced Glutathione Synthesis. *Chem Res Toxicol*. **2019**;32(3):421-436. doi:10.1021/acs.chemrestox.8b00259

S35. Chang WJ, Chen BH, Inbaraj BS, Chien JT. Preparation of allyl isothiocyanate nanoparticles, their anti-inflammatory activity towards RAW 264.7 macrophage cells and anti-proliferative effect on HT1376 bladder cancer cells. *J Sci Food Agric*. **2019** Apr;99(6):3106-3116. doi: 10.1002/jsfa.9524.

S36. Campbell JK, Canene-Adams K, Lindshield BL, Boileau TW, Clinton SK, Erdman JW Jr. Tomato phytochemicals and prostate cancer risk. *J Nutr*. **2004**. 134(12 Suppl):3486S-3492S. doi: 10.1093/jn/134.12.3486S.

S37. Applegate CC, Lowerison MR, Hambley E, Song P, Wallig MA, Erdman JW Jr. Dietary tomato inhibits angiogenesis in TRAMP prostate cancer but is not protective with a Western-style diet in this pilot study. *Sci Rep*. **2021**. 17;11(1):18548. doi: 10.1038/s41598-021-97539-2.

S38. Kim MJ, Kim H. Anticancer Effect of Lycopene in Gastric Carcinogenesis. *J Cancer Prev*. **2015**;20(2):92-96. doi:10.15430/JCP.2015.20.2.92

S39. Linnewiel-Hermoni K, Khanin M, Danilenko M, et al. The anti-cancer effects of carotenoids and other phytonutrients resides in their combined activity. *Arch Biochem Biophys***. 2015**;572:28-35. doi:10.1016/j.abb.2015.02.018

S40. Le V, Esposito D, Grace MH, et al. Cytotoxic effects of ellagitannins isolated from walnuts in human cancer cells. *Nutr Cancer*. **2014**;66(8):1304-1314. doi:10.1080/01635581.2014.956246

S41. Hardman WE. Walnuts have potential for cancer prevention and treatment in mice. *J Nutr*. **2014**;144(4 Suppl):555S-560S. doi:10.3945/jn.113.188466

S42. Catanzaro E, Greco G, Potenza L, Calcabrini C, Fimognari C. Natural Products to Fight Cancer: A Focus on *Juglans regia*. *Toxins (Basel).* **2018**. 14;10(11):469. doi: 10.3390/toxins10110469.

S43. Bae H, Song G, Lim W. Stigmasterol Causes Ovarian Cancer Cell Apoptosis by Inducing Endoplasmic Reticulum and Mitochondrial Dysfunction. *Pharmaceutics*. **2020**;12(6):488. doi:10.3390/pharmaceutics12060488

S44. Sajadimajd S, Yazdanparast R, Roshanzamir F. Augmentation of oxidative stress-induced apoptosis in MCF7 cells by ascorbate-tamoxifen and/or ascorbate-juglone treatments. *In Vitro Cell Dev Biol Anim*. **2016;**52(2):193-203. doi:10.1007/s11626-015-9961-4

S45. Prasad S, Tyagi AK. Ginger and its constituents: role in prevention and treatment of gastrointestinal cancer. *Gastroenterol Res Pract*. **2015**;2015:142979. doi:10.1155/2015/142979

S46. Parham S, Kharazi AZ, Bakhsheshi-Rad HR, Nur H, Ismail AF, Sharif S, RamaKrishna S, Berto F. Antioxidant, Antimicrobial and Antiviral Properties of Herbal Materials. *Antioxidants (Basel).* **2020**. 21;9(12):1309. doi: 10.3390/antiox9121309.

S47. Jiang X, Wang J, Chen P, et al. [6]-Paradol suppresses proliferation and metastases of pancreatic cancer by decreasing EGFR and inactivating PI3K/AKT signaling. *Cancer Cell Int*. **2021**;21(1):420. doi:10.1186/s12935-021-02118-0

S48. Zhang, MM., Wang, D., Lu, F. *et al.* Identification of the active substances and mechanisms of ginger for the treatment of colon cancer based on network pharmacology and molecular docking. *BioData Mining*. **2021.** 14, 1. doi.org/10.1186/s13040-020-00232-9

S49. Jeong JH, An JY, Kwon YT, Rhee JG, Lee YJ. Effects of low dose quercetin: cancer cell-specific inhibition of cell cycle progression. *J Cell Biochem*. **2009**;106(1):73-82. doi:10.1002/jcb.21977

S50. Adams LS, Phung S, Yee N, Seeram NP, Li L, Chen S. Blueberry phytochemicals inhibit growth and metastatic potential of MDA-MB-231 breast cancer cells through modulation of the phosphatidylinositol 3-kinase pathway. *Cancer Res*. **2010**;70(9):3594-3605. doi:10.1158/0008-5472.CAN-09-3565

S51. Kanaya N, Adams L, Takasaki A, Chen S. Whole blueberry powder inhibits metastasis of triple negative breast cancer in a xenograft mouse model through modulation of inflammatory cytokines. *Nutr Cancer*. **2014**;66(2):242-248. doi:10.1080/01635581.2014.863366

S52. Thibado SP, Thornthwaite JT, Ballard TK, Goodman BT. Anticancer effects of Bilberry anthocyanins compared with NutraNanoSphere encapsulated Bilberry anthocyanins. *Mol Clin Oncol*. **2018**;8(2):330-335. doi:10.3892/mco.2017.1520

S53. Adams LS, Phung S, Yee N, Seeram NP, Li L, Chen S. Blueberry phytochemicals inhibit growth and metastatic potential of MDA-MB-231 breast cancer cells through modulation of the phosphatidylinositol 3-kinase pathway. *Cancer Res*. **2010**;70(9):3594-3605. doi:10.1158/0008-5472.CAN-09-3565

S54. Adams LS, Kanaya N, Phung S, Liu Z, Chen S. Whole blueberry powder modulates the growth and metastasis of MDA-MB-231 triple negative breast tumors in nude mice. *J Nutr*. **2011**;141(10):1805-1812. doi:10.3945/jn.111.140178

S55. Li L, Adams LS, Chen S, Killian C, Ahmed A, Seeram NP. Eugenia jambolana Lam. berry extract inhibits growth and induces apoptosis of human breast cancer but not non-tumorigenic breast cells. *J Agric Food Chem*. **2009**;57(3):826-831. doi:10.1021/jf803407q

S56. Sánchez C. Reactive oxygen species and antioxidant properties from mushrooms. *Synth Syst Biotechnol*. 2016;2(1):13-22. Published 2016 Dec 24. doi:10.1016/j.synbio.2016.12.001

S57. Oba K, Kobayashi M, Matsui T, Kodera Y, Sakamoto J. Individual patient based meta-analysis of lentinan for unresectable/recurrent gastric cancer. *Anticancer Res*. 2009;29(7):2739-2745.
